# Opinion: How does XIST promote sex bias in autoimmune diseases?

**DOI:** 10.3389/fimmu.2024.1399408

**Published:** 2024-04-11

**Authors:** Felipe Andrade

**Affiliations:** Division of Rheumatology, The Johns Hopkins University School of Medicine, Baltimore, MD, United States

**Keywords:** XIST, lupus, autoimmune, toll-like receptor, autoantibodies, interferon, sex-biased

The risk of autoimmunity linked to female sex is substantially larger than any susceptibility gene discovered to date (1). The mechanisms underlying sex bias in autoimmune diseases, however, remain poorly understood. By approaching different hypotheses, two recent studies reached to the conclusion that X-inactive-specific transcript (XIST) – a X chromosome-encoded long noncoding RNA (lncRNA) – may explain sex bias in systemic lupus erythematosus (SLE) and potentially other female-associated autoimmune diseases (2, 3). Because the function of XIST is to inactivate one of the two X chromosomes in females (4), it is not expressed in males, explaining why XIST is a strong candidate for underlying female predominance in autoimmune diseases. Interestingly, far from its function in X chromosome inactivation (XCI), both studies suggest that XIST rather induces autoimmunity by activating innate or adaptive immune responses (2, 3). While both studies complement one other, the proposed mechanisms are different, which merit an examination of their discrepancies.

Because gain-of-function of toll-like receptor 7 (TLR7), a cellular sensor of viral infection activated by RNA, is a cause of SLE (one of the most female sex-biased autoimmune diseases) (5), Crawford et al., used unbiased approaches to search for endogenous female-specific TLR7 ligands (2). Using differential expression analysis of peripheral blood between female and male donors, as well as transcriptional data from SLE tissues (i.e., blood, spleen, and kidney), they ranked transcripts based on 4 criteria: female expression bias, total UU count, maximum UU richness, and expression. Remarkably, this approach led to the discovery of XIST as the strongest candidate carrying female-specific TLR7 ligands in the entire human genome. Further studies demonstrated that XIST, but not other RNAs in the cellular transcriptome, activates TLR7 triggering the production of interferon (IFN)-α by plasmacytoid dendritic cells, which is a hallmark in SLE (6). Notably, XIST expression was found to be increased in leukocytes from women with SLE, which correlated with disease activity and the IFN signature. Moreover, they showed that XIST is not IFN inducible, implying that XIST is a cause rather than a result of IFN production in SLE.

In contrast to the work by Crawford et. al., which indicates that XIST drives autoimmunity by directly acting as a DAMP (Damage-Associated Molecular Pattern) (2), Dou et al., proposed that XIST plays an indirect role in autoimmunity by providing protein autoantigens to the adaptive immune system (3). Since XIST is a ribonucleoprotein (RNP) enriched with protein antigens targeted in autoimmune diseases, Dou et. al., hypothesized that XIST may promote female-biased autoimmunity by serving as an autoantigen carrier (3). Relevant to this model, however, it is important to note that autoantigens found on XIST are not X-linked or XIST-specific, but rather found in a range of ubiquitously expressed non-sex-biased nucleoproteins targeted in autoimmune diseases (e.g., Ro, La, spliceosomes, HMGB1, TIF1-γ, PARP1, etc.). In addition, autoantibodies to the X inactive chromosome (i.e., Barr body), where XIST is localized (7, 8), are extremely rare in autoimmune diseases (i.e., 0.004 to 0.0054%) (9, 10).

To study the role of XIST in autoantibody production and autoimmunity, Dou et. al., generated male transgenic mice expressing an inducible non-silencing form of XIST, which has no effect on XCI but retains binding to proteins (3), and challenged these mice with pristane to induce lupus. Important to this model, pristane-induced lupus is female-biased and completely dependent on TLR7 activation (11–13). Interestingly, unlike wild-type male mice, two-thirds of males expressing XIST developed severe multi-organ disease after pristane injection, which was comparable to pristane-induced lupus in females, demonstrating that XIST overexpression predisposes to autoimmune disease development. The disease, however, was only induced in autoimmune prone male SJL/J mice but not in C57BL/6J male mice, implying that XIST overexpression alone is insufficient to promote autoimmunity in this experimental model. Considering that the transgene was introduced into an autosome and has no effect on XCI, it is unclear why XIST overexpression was not addressed in female mice challenged with pristane.

Different to SLE, pristane-induced lupus is independent of the production of anti-DNA antibodies. Instead, the disease is caused by antibodies to RNPs, which is explained given that pristane-induced lupus is TLR7-driven and negatively regulated by TLR9 (14). Consistent with this model, male SJL/J mice expressing transgenic XIST developed antibodies to RNPs following injection with pristane (3). However, while the protein targets of these antibodies, as well as autoantibodies found in autoimmune diseases in humans, are components of the XIST RNP (3), they are not XIST-specific. Therefore, it is difficult to elucidate whether the induction of lupus in transgenic XIST male mice is caused by an increase in autoantigen load, as concluded by Dou et al. (3), or by simply driving TLR7 activation, as previously demonstrated by Crawford et. al., in the human model (2).

While the induction of lupus in transgenic XIST male mice is enticing, it is important to evaluate the significance of this model in the context of autoimmune diseases in humans. First, the limited immunogenicity of Barr bodies in autoimmune diseases (9, 10) challenges the notion that XIST RNP drives autoimmunity in humans by acting as an autoantigen. Second, a hallmark feature in human autoimmune diseases is the precise association of clinical phenotypes [e.g., SLE, scleroderma, and rheumatoid arthritis (RA)] with unique autoantibody specificities (e.g., Sm/nucleosomes, topoisomerase I, and citrullinated antigens, respectively) (15). The notion that XIST RNP acts as an autoantigen to induce autoimmunity implies that all female-associated autoimmune diseases are caused by the production of autoantibodies to XIST RNP, regardless of the disease phenotype. Aside from questioning the importance of autoantigen specificity in autoimmune disease pathogenesis, this model makes it difficult to explain, for example, how an initial antibody response to XIST RNP will drive the production of antibodies to citrullinated antigens in RA or anti-dsDNA antibodies in SLE years before the onset of disease. The idea that XIST predisposes to autoimmune diseases by mirroring signals activated by viruses, such as acting as a DAMP on TLR7 (2), is appealing because it can create a non-specific pro-inflammatory milieu that may allow for additional autoantigen-specific immune responses in individual autoimmune diseases. Defining the role of TLR7 in the induction of lupus in male SJL/J mice expressing transgenic XIST is therefore important for determining whether this mouse model is equivalent to the autoimmune model in humans.

## Author contributions

FA: Conceptualization, Funding acquisition, Investigation, Writing – original draft, Writing – review & editing.
